# Genome-wide Association Study Meta-analysis of Blood Pressure Traits and Hypertension in Sub-Saharan African Populations: An AWI-Gen Study

**DOI:** 10.21203/rs.3.rs-2532794/v1

**Published:** 2023-02-13

**Authors:** Surina Singh, Ananyo Choudhury, Scott Hazelhurst, Nigel Crowther, Palwende Boua, Hermann Sorgho, Godfred Agongo, Engelbert Nonterah, Lisa Micklesfield, Shane Norris, Isaac Kisiangani, Shukri Mohamed, Francesc Gomez-Olive, Stephen Tollman, Solomon Choma, Jean-Tristan Brandenburg, Michele Ramsay

**Affiliations:** Sydney Brenner Institute for Molecular Bioscience (SBIMB), University of the Witwatersrand; University of the Witwatersrand; Sydney Brenner Institute for Molecular Bioscience, Faculty of Health Sciences & School of Electrical & Information Engineering, University of the Witwatersrand; 11Department of Chemical Pathology, National Health Laboratory Service; Clinical Research Unit of Nanoro, Institut de Recherche en Sciences de la Santé; Clinical Research Unit of Nanoro, Institut de Recherche en Sciences de la Santé; NavrongoHealth Research Centre, Navrongo, Ghana; Navrongo Health Research Centre, Ghana Health Service; University of the Witwatersrand; SAMRC Developmental Pathways For Health Research Unit, Department of Paediatrics & Child Health, University of the Witwatersrand, Johannesburg, South Africa; African Population and Health Research Center; African Population and Health Research Center; 8MRC/Wits Rural Public Health and Health Transitions Research Unit (Agincourt), School of Public Health, Faculty of Health Sciences, University of the Witwatersrand; University of the Witwatersrand; Department of Pathology and Medical Sciences; Sydney Brenner Institute for Molecular Bioscience (SBIMB), University of the Witwatersrand; University of the Witwatersrand

## Abstract

Most hypertension-related genome-wide association studies (GWAS) focus on non-African populations, despite hypertension (a major risk factor for cardiovascular disease) being highly prevalent in Africa. The AWI-Gen study GWAS meta-analysis for blood pressure-related traits (systolic and diastolic blood pressure, pulse pressure, mean-arterial pressure and hypertension) from three sub-Saharan African geographic regions (N=10,775), identified two genome-wide significant signals (p<5E-08): systolic blood pressure near P2RY1 (rs77846204; intergenic variant, p=4.25E-08) and pulse pressure near Linc01256 (rs80141533; intergenic variant, p=4.25E-08). No genome-wide signals were detected for the AWI-Gen GWAS meta-analysis with previous African-ancestry GWASs (UK Biobank (African), Uganda Genome Resource). Suggestive signals (p<5E-06) were observed for all traits, with 29 displaying pleiotropic effects and several replicating known associations. Polygenic risk scores developed from studies on different ancestries had limited transferability, with multi-ancestry models providing better prediction. This study provides insights into the genetics and physiology of blood pressure variation in African populations.

## Introduction

Hypertension (HTN) is a major risk factor for cardiovascular diseases (CVD) such as coronary heart disease, heart valve diseases, atrial fibrillation, aortic syndromes, cerebral stroke and renal failure^1,2^ Between 1990 and 2019, hypertension prevalence almost doubled for adults (aged 30–79 years) and affected 1.25 billion people living in low- and middle-income countries^3^. This increase is attributed to population growth and ageing, and is predicted to increase to 1.56 billion people by 2025^4,5^ in addition, HTN is a leading risk factor for premature deaths and disability worldwide^6,7^, accounting for 17.9 million deaths in 2018^8^. it is present in ~22% of the global population, with the highest prevalence observed in Africa (27%), particularly in urban communities and in older people^9^.

HTN prevalence and awareness differs between and within the sub-Saharan African countries^10^. There is a paucity of data on the prevalence, treatment and control of HTN in many African countries and therefore its contribution to related conditions, such as hypertrophic cardiomyopathy, is not fully understood^11,12^. Major research focuses on genetic associations with HTN, due to its high prevalence and the fact that it doubles the risk for CVD^8,13^. Familial studies have shown HTN associations amongst immediate family members, with genetic factors explaining approximately 30–50% of blood pressure (BP) variation amongst individuals^14,15^. However, these studies have limitations in identifying genetic variants responsible for increased risk of developing HTN.

Genome-wide association studies (GWASs) have explained only a modest proportion of the genetic heritability (3–6%) for blood pressure (BP) and HTN^16^. The GWAS Catalog^17 18^ includes data from the first BP/HTN case-control studies conducted in 2007 for HTN^19^ and for BP as a quantitative trait^20^. The GWAS Catalog currently includes 7,982 genetic associations with BP based on 380 studies and 586 associations with HTN based on 120 studies (https://www.ebi.ac.uk/gwas, accessed 17 November 2022).

Early GWAS studies outlined the complexity of studying BP-related traits and emphasised the importance of large sample sizes to enable the detection of genetic associations^19,20^. Large-scale GWAS discovery meta-analyses have shown significant genetic associations with BP and HTN^16,21,22^. The largest BP GWAS to date by Evangelou, et al.^22^ included over 1 million individuals of European ancestry from the UK Biobank (UKBB) and the International Consortium of Blood Pressure (ICBP), identifying 535 BP-related loci.

Only a small number of GWAS for genetic associations with BP and HTN have been performed on the African continent. Despite HTN being highly prevalent in Africa^23^, most studies have focused on the European populations^16^. Studies based on African-ancestry populations comprise mainly of African American (AA) populations^24–28^, with the first GWAS for HTN in AA conducted in 2009 by Adeyemo, et al.^29^. Hendry, et al.^30^ studied a black South African population (n = 1947 with ~700 women who are also present in our study) with samples genotyped using the Metabochip (~200,000 single nucleotide polymorphisms (SNPs) previously associated with cardiometabolic traits). They found genetic associations with systolic and diastolic BP in genes of interest *(NOS1AP, MYRF and POC1B)* and in some intergenic regions (*DACH1/LOC440145* and *INTS10/LPL)*^30^.

African populations have high genetic diversity, allele frequency differences and low linkage disequilibrium (LD) when compared to other populations^31^ and therefore GWASs from sub-Saharan Africa have the potential to discover novel BP-related SNPs. However, it is important to recognise and adjust for extensive population structure across different African regions^32,33^ in genetic association studies and to use genotyping arrays such as the Human Heredity and Health in Africa (H3Africa) SNP array, that is enriched for common genetic variants in African populations^34^.

In this study, the sub-Saharan African cohort of older adult participants, referred to as the Africa Wits-INDEPTH partnership for Genomic Studies (AWI-Gen)^35,36^, was used and DNA samples genotyped with the H3Africa SNP array. The aim of the study was to identify genetic associations with four continuous BP-related traits (systolic BP (SBP), diastolic BP (DBP), pulse pressure (PP) and mean-arterial pressure (MAP)) and one categorical trait (HTN), in three sub-Saharan African regions represented in the AWI-Gen study. To boost power, the findings were meta-analyzed with other studies the included African or African-ancestry participants. Fine mapping and genetic risk score analysis and transferability were also assessed.

## Results

Participants in the AWI-Gen cohort had a mean age (SD) of 51.8 (8.2) years, with slightly more women (54.7%). The average BMI of the cohort was 25.1 (6.7) kg/m^2^ (defined as overweight (body mass index (BMI) between 25.0 to 29.9 kg/m^2^). The average resting heat rate was within the normal range (<100 beats per minute). The majority of study participants fell within the normal to pre-HTN BP category (126.9/83mmHg; 3818 HTN cases, 6918 HTN controls), with most self-reported as not using anti-hypertension medication (AHM) (76.8%) (Table 1). Among individuals identified as having HTN, more had stage 1 (17.2%), were not using AHM (16.3%), didn’t have parents with HTN (12.7%) and were un-aware of their HTN status and not controlling for HTN (11.5%) (Table S 2). The discovery GWASs for the five BP traits (SBP DBP, HTN, PP, MAP) was conducted on 10,700 sub-Saharan African participants with 13,976,041 SNPs. For each BP-related trait, quality control (QC) was performed and adjustments were made for the use of AHM (Figure S 1). The power calculation revealed that the current study has at least 80 % power to detect an effect size beta of ~0.60 for SNPs with MAF>0.10 (Figure S 3).

### Genetic associations with BP traits

Association studies were performed in two stages: Stage 1 – meta-analysis of the GWAS for the three geographic regions represented in the AWI-Gen cohort (N=10,775); Stage 2 – Meta analysis of Stage 1 with GWAS from other studies on African and African-ancestry populations (UKBB African-ancestry (UKBBa, N=3,058) and Uganda Genome Resource (UGR, N=6,400)) (Figure 2). Genetic associations with each of the five BP traits are shown, using Miami plots and genomic inflation by the QQ-plots, for both Stage 1 and 2. There was no indication of genomic inflation, since the genomic inflation factor (GIF), lambda (λ), was <1.05 for all five BP traits, indicating adequate control for population sub-structure (Figure S 2). Independent GWASs for each AWI-Gen region (East, West, and South) were also conducted (Figure S 4). When comparing Stage 1 and Stage 2 GWASs for SBP and DBP (Figure 2), the signals differed by region.

Prior to the Stage 1 meta-analyses, genome-wide (GW) associations for 41 SNPs with 9 displaying pleiotropic effects at GW significance were found in the three independent AWI-Gen regions (Table S 3). Thus, 12 signals each for East (2 displaying pleiotropic) and South (4 pleiotropic), with 5 and 11 SNPs reaching suggestive significance (p<5E-06) respectively, were observed. For West Africa 14 signals (2 pleiotropic) with only six reaching suggestive significance (Table S 4) were identified. Due to regional differences, the mega-analyses (a single GWAS for the entire AWI-Gen study for each trait) when compared to the AWI-Gen meta-analyses gave different GW associations with different associations identified across regions (Figure S 4). To account for the diverse signals driven by each independent region (regional differences), a meta-analysis of the three independent region GWASs was done using Han-Eskin random effects (RE2) model (Stage 1).

## Stage 1 GWAS

Suggestive associated genomic regions (p<1E-06) from the Stage 1 discovery GWAS (identified in FUMA), are shown in Table 2. Across the five traits, 129 independent genomic regions were identified (136 independent SNPs with 130 lead SNPs), with 29 genomic region signals associating with more than one BP-trait (see bold SNPs in Table S 4), illustrating pleiotropic effects. When comparing the Stage 1 GWAS by region, suggestive signals (p<5E-04) differed across the East, West and South African regions (Table S 4).

The GW significance threshold (p<5E-08) was reached for SBP with rs77846204 (imputed intergenic variant in *RP11–38P22.2*, p=4.95E-08) (Table 2), driven by the West (p=4.16E-07) and East (p=3.24E-04) AWI-Gen regions (Table S 4). This signal displayed pleiotropic effects with DBP (p=1.66E-06) and MAP (p=1.51E-07) (see bold SNPs in Table S 4) and had a high allele frequency in previous studies for all ancestries (>0.8, Ancestries: African, Admixed American, East Asian, European, African Americans). GW significance was also reached for PP with rs115808349 (imputed intergenic variant in *ELL2P2*, p=1.76E-08), driven by the East AWI-Gen region (p=2.25E-05) (Table S 4) and had low allele frequency for all ancestries, with African being the highest (0.05).

Several suggestive independent genomic regions (Table 2) were observed across the five BP-related traits (40 SBP, 25 DBP, 21 HTN, 33 PP, and 31 MAP). The strongest signals (lowest p-values) for traits that reached only suggestive significance were: DBP, rs6494981 (intergenic *TMCO5B*, p=9.40E-08), which was also a suggestive signal for MAP (p=1.59E-06, displaying pleiotropic effects); HTN, rs113112741 (intronic *MAML3*, p=7.12E-08); MAP, rs73315125 (intergenic *FDPSP6*, p=8.02E-08), which was also a suggestive signal for SBP (p=6.35E-08) and DBP (p=1.13E-06, displaying pleiotropic effects).

## Stage 2 GWAS

The number of SNPs increased from 13,952,382 (Stage 1) to 14,845,228 for the Stage 2 GWAS. No GW associations were detected in the Stage 2 analysis for any of the traits. Stage 2 GWAS suggestive independent genomic regions (p<1E-06), identified in FUMA, are shown in Table 3 with 40 independent genomic regions (17 SBP, 23 DBP (42 independent SNPs with 40 lead SNPs)). Most of these signals were driven by the Uganda Genome Resource (UGR) dataset (p<5E-04, Table S 5). Only one SBP (rs17428471) and five DBP (rs114007149, rs141245590, rs474277, rs617549, rs556594) signals were also identified in the Stage 1 GWAS, reaching suggestive significance. The signals with the lowest p-values for traits that reached only suggestive significance were: SBP, rs115702999 (ncRNA_exonic *HECW2:AC020571.3*, p=2.77E-07); DBP, rs6009081 (intronic *PPARA*, p=5.75E-07). Pleiotropic effects were not observed.

### Replication of Stage 1 and 2 GWAS outcomes

Exact replication was conducted by comparing GW SNPs (p<5E-08) with suggestive SNPs (p<5E-04) from this study (Stage 1 and Stage 2) with GW SNPs from previous BP GWASs^17,22,37,38^. No replication of the two GW SNPs (p < 5E-08) for SBP (rs77846204, beta=17.7, p=4.95E-08) and PP (rs115808349, beta=32.4, p=1.76E-08) were found when compared against suggestive SNPs of the previous BP GWASs (p < 5E-04). Both GW signals were novel to this study.

This study identified 25,338 SNPs (12,385 pleiotropic SNPs) across the five BP-related traits for Stage 1 (SBP, DBP, HTN, PP, MAP) and 10,632 SNPs (532 pleiotropic SNPs) for Stage 2 (SBP, DBP) that met replication suggestive significance (p<5E-04), with only 217 SNPs in both Stages. Replication of only 596 GW significant SNPs within 132 identified genomic regions (p<5E-08) from previous studies were found (503 Stage 1, 115 Stage 2, 23 both Stages) when compared against suggestive SNPs (p<5E-04) of the Stage 1 and Stage 2 GWASs (Table 4). Replication for each previous study is reported i.e. GWAS Catalogue^17^, Warren, et al.^38^ and Evangelou, et al.^22^ (Table S 6). Several SNPs illustrating pleiotropic effects were identified, with most replicated SNPs from European-ancestry studies. Three replicated SNPs (Stage 2) were from trans-ethnic studies that included African ancestry participants and displaying pleiotropic effects i.e. rs9821489^39^(SBP, DBP), rs17428471^25,40^ (SBP,DBP,PP, BP), and rs891511^41,42^ (SBP, DBP, HTN, MAP).

### Fine-mapping and functional analysis

Fine-mapping, to identify potential causal variants, for GW SNPs (p<5E-8) and/or top signal SNPs are shown as regional plots in Figure 3 and Figure S 5 respectively. Potential causal variants that met GW significance were identified for SBP (rs77846204, p=4.95E-08) and PP (rs115808348, p=1.76E-08), for the Stage 1 GWAS (Figure 3). The top signal SNPs (lowest p-value) for BP traits that only met suggestive significance for both the Stage 1 and 2 GWASs (p<5E-06) can be seen in Figure S 5, with none replicating (Figure S 5).

For SBP the regional plot around the *P2RY1* region for rs77846204 (chr3, p=4.95E-08, intergenic *RP11–38P22.2*) showed a very narrow peak (Figure 3), with an allele frequency of 0.81 for African populations (Table S 4). This SNP was found to be closely associated with SBP (p = 2.00E-08) and PP (p = 4.00E-11) for rs161792 (distance = 0.40MB) and with PP only for rs325729 (p=9.00E-09, distance = −0.44MB) and rs146975914 (p=5.00E-09, distance = −0.32MB) in other studies^39,43,44^ (Table S 7). It was also associated with other CVD linked traits such as HDL cholesterol, lung function/post bronchodilator (FEV1, associated with lung function), liver function tests and type 2 diabetes. It was also closely related to prostate cancer, baldness/hair colour, white cell counts and mood-related traits, within 1MB flanking region.

For PP the regional plot around the *Linc01256* region for rs115808349 (chr4, intergenic *ELL2P2)*, showed two narrow peaks within the same region (Figure 3), since it also consisted of rs62317311 (chr 4, ncRNA_intronic *RP11–789C2.1*), that only reached suggestive significance (p=8.92E-07). This SNP had the highest allele frequency (0.05) for African population, with extremely low allele frequencies (<0.004) found in non-African populations (Table S 4). This SNP was found to be closely associated with resistance to AHM in HTN for rs1908127 (p=2.00E-06, distance = 0.15MB) (Table S 7). This SNP was found to be closely associated with traits such as total PHF-tau (SNP × SNP interaction), protein quantitative trait loci (liver) and mood-related traits within 1MB flanking region.

Annotation of variants with GW significance (p<5E-08) for SBP (rs77846204) and PP (rs115808349), showed that these were intergenic (Table S 8). None of the SNPs were potentially deleterious (CADD score <12.37), with the exception of rs116166107 for PP (CADD = 12.72). Genetic positions for SBP (rs77846204) and PP (rs11580834) had a RDB score of 6 and 7 respectively. Functional mapping of position, eQTL (matched cis-eQTL SNPs) and chromatin interaction (i.e. 3D DNA–DNA interactions) are reported in Table S 9.

### PRS

Polygenic risk scores (PRS), developed from three ancestry (African, European and multi-ancestry) GWASs (discovery) were applied to the individuals in AWI-Gen cohort (target, N=10,676) for SBP and DBP (shown in Figure 4).

All PRSs developed from the different ancestries, show an increase in predicting higher BP levels as the quantile score increases (Figure 4a). The highest change in effect size (mm/Hg) was observed in the model from the multi-ancestry population, whereas the lowest change was observed in the African-ancestry PRS derived from the UKBBa dataset for both SBP and DBP.

The variance explained i.e. R2 (%), was highest for the multi-ancestry PRS (0.22% for SBP and 0.36% for DBP) and lowest for the African-ancestry PRSs for both SBP (for UKBBa: 0.07%) and DBP (for UGR: 0.04%) (Figure 4b). The PRS was significant (p<P-threshold (PT)) for SBP in UGR African-ancestry, for DBP in European-ancestry and for both SBP and DBP in UKBBa and multi-ancestry, indicating transferability. The multi-ancestry models had the highest number of SNPs for SBP (N = 326,601) and second highest for DBP (N = 69071).

## Discussion

HTN is a complex multifactorial disease that involves interactions of multiple variants in many genes, together with environmental risk factors^15,45^, thereby making the identification of genetic associations complicated. AWI-Gen is a population-based cross-sectional cohort from Africa with a high prevalence of HTN, showing low awareness and control of high BP and control, suggesting a lack of effective treatment^10^.

The strengths of this study include following the same standard procedures and analyses parameters for all AWI-Gen participants across the different geographic regions of Africa and the same genotype array and imputation panels, for a GWAS performed in a population with a high prevalence of HTN. The AWI-Gen discovery GWAS is based on participants from three different African regions that exhibit significant population structure and previous analyses identified the presence of cryptic relatedness in some subsets, requiring adjustments during the analysis. To address this limitation, three region-based GWASs were conducted for the East, South and West African regions and meta-analysed for the AWI-Gen dataset (Stage 1 GWAs). The dataset was of modest sample size, with limited power to identify associations with markers with low minor allele frequency and small effect sizes. To address this limitation, at least partially, the AWI-Gen data was meta-analyzed with published summary statistics from GWAS performed on African and African-ancestry cohorts. Availability of suitable African datasets to replicate the novel associations was a major challenge. Therefore, replication was conducted using both modest African datasets and large European datasets. Finally, the comprehensive evaluation of risk models was dependent on the availability of a suitable independent African dataset, with the UGR cohort^46^ and UKBBa being the most closely related datasets available.

Several suggestive signals (p<5E-06) were identified in the Stage 1 and 2 GWAS (Table 2), with the identification of two novel SNPs that reached GW significance for SBP in the Stage 1 discovery GWAS. Regional GW associations across Africa were observed in this study (Table S 3). The lack of multiple large African-ancestry datasets and diversity within each African region could contribute to the absent replication of the meta-analysis with larger African-ancestry populations. Currently most African GWAS studies are limited to cohorts from Uganda, Nigeria and South Africa and studies that include admixed AA populations^47^. There is a need for larger GWAS of continental African populations, to better investigate the role of these SNPs in BP regulation among Africans. Similar to this study, genetic associations linked with BP-related traits in African populations have been found to be limited to those populations^25,26,47,48^.

In contrast to this study’s discovery GWAS, the GWASs of both the UKBBa and UGR^46^ cohorts did not make adjustments for AHM. The UGR cohort also did not include the first 10 principal components (PCs) as covariates and performed an inverse normal transformation. Other potential African studies had to be excluded, due to the lack of data availability, diversity of populations or pooled admixed African-ancestry datasets^47^. Some datasets had large proportions of participants with traits that could potentially influence HTN status and were therefore excluded from the meta-analyses (these included the Africa America Diabetes Mellitus Study (AADM), Durban Case Control Study (DCC) and Durban Diabetes Study (DDS) cohorts each with ~50% diabetic participants^46^). Having diabetes could affect BP since it causes the walls of the blood vessels to stiffen which could lead to HTN, with many studies reporting a correlation between diabetes and HTN^49–51^. The lack of knowledge of co-factors, such as diabetes (for prevalence and medication used) not being recorded, is cause for concern when trying to determine genetic association with HTN^49^. Failure to replicate SNPs that reached GW significance in this study could also be due to small sample sizes, which would affect the power of each study to detect associations. In addition, protocols for phenotype (measurement errors) and genotype (array and imputation panels used) data, allele frequency, LD (variants with the causal SNPs) and effect sizes (attributed gene-environmental interactions) may also differ^46,47^. The low replication rate of GW associations (Table 4) found in European studies may be attributed to the low power of the small studies to detect small effect variants. The smaller sample size for African-ancestry studies are only powered to detect large effect associations^47^.

Similar to this study, several BP-related traits are associated with the same SNPs, displaying pleiotropic effects, and are mapped to non-coding genomic regions, making functional mapping challenging^52^. Blood pressure multi-trait analyses, using the SHet and SHom approaches (which account for the correlation of the multi-traits and for overlapping or related samples among the cohorts), provide greater power to detect BP associations with the SHet method resulting in more associations compared to SHom^26,53^. Blood pressure-related traits are closely linked with other CVD risk factors such as insulin resistance, obesity, kidney function, atherogenic dyslipidaemia, stroke and coronary artery disease^54^. Heterogenous regional patterns and associations have been identified between HTN and obesity within continental Africans^55^. The influence of these CVD risk factors on BP-related traits, along with other gene-gene, gene-environment, and lifestyle effects among different geographic regions within Africa, could limit the relatively small AWI-Gen sample population’s ability to accurately identify genetic associations and should be further investigated.

Since an individual’s genetic data is stable throughout life, it can be used to potentially predict future disease risk. The PRSs derived from multi-ancestry populations provided higher risk prediction of BP-related traits in the AWI-Gen cohort compared to PRSs from other African ancestry populations (Figure 4). In contrast, Choudhury, et al.^56^ and Kamiza, et al.^57^ found that PRSs derived from African ancestry populations had higher risk prediction for lipid traits in sub-Saharan Africans compared to European and multi-ancestry scores. This shows that BP-related traits are more complex to understand and the low transferability of PRS models to Africans could be attributed to the smaller sampler size, differences in LD, allele frequency and gene-environmental factors. Poor transferability has been demonstrated for existing European-based PRS models to African ancestry populations for most phenotypes^4,56–59^. This stresses the importance for further research to optimize PRS prediction in non-European populations, specifically African populations, with increased sample sizes to enhance PRS prediction^56,58^. Machine learning, using GWAS summary statistics, was found to improve the risk prediction of PRSs for traits that influence the risk of HTN (diabetes, obesity/BMI and height) and this suggests that it may also enhance PRSs for hypertension^60,61^. The lack of interpretability of machine learning encourages the development of hybrid techniques, such as combining Mendelian randomization with machine learning post-GWAS approaches, to identify causal inference of associated variants^62,63^.

Future GWASs should focus on regional differences in continental Africa with large sample sizes per region to better understand these associations. The BP-related traits analysed in this study display pleiotropic effects that should be explored further, along with conducting BP multi-trait analyses to increase study power^26,53^. Gene-gene and gene-environment interactions of BP-related traits should also be explored to better understand genetic heritability (where only 3–6 % can currently be explained by GWASs)^16^. Pharmacogenomics studies that focus on drug–gene interactions and treatment outcome may lead to improved clinical treatment guidelines^52^.

## Conclusion

Two signals of GW significance were observed in a discovery (Stage 1) GWAS for SBP and PP with no GW significant associations detected in a meta-analysis (Stage 2). Several suggestive signals were observed for all traits in both Stages, with 29 associations displaying pleiotropic effects and several replicating known associations. Limited transferability was observed from PRSs developed from the different ancestries, with the best prediction using a multi-ancestry model. The identification of new genetic associations with BP-related traits, as investigated in this study, will contribute to understanding the genetic aetiology of BP variation in African populations and could help provide additional biological insights.

## Methods

**Figure 1:**
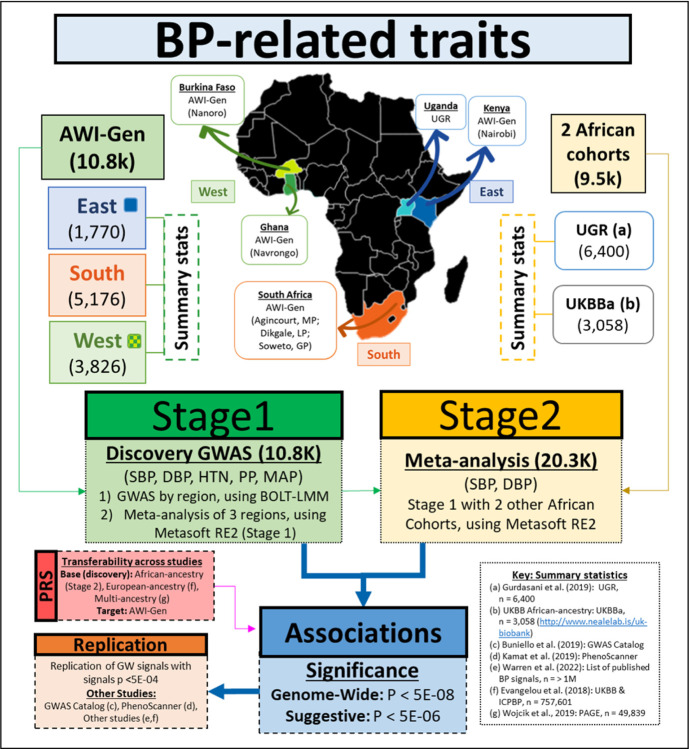
Workflow summary for datasets and analyses. Stage 1 GWAS conducted for all five BP-related traits i.e. a meta-analysis of each AWI-Gen region (East, South, West) per trait – sample sizes are indicated in brackets. Stage 2 GWAS conducted for SBP and DBP i.e. a meta-analysis of the Stage 1 results with other African-ancestry summary statistics i.e. UGR 46 and UKBB. Replication of associations were assessed using the GWAS Catalog^17^, PhenoScanner^37^ and other studies summary statistics^22,38^. Transferability across studies was conducted via PRS models based on African-ancestry (UGR, UKBBa), Multi-ancestry i.e. PAGE^41^ and European-ancestry i.e. UKBB & ICPBP^22^ cohorts (discovery) used to assess the distribution of SBP and DBP risk according to PRS deciles in the AWI-Gen cohort (target).

**Figure 2:**
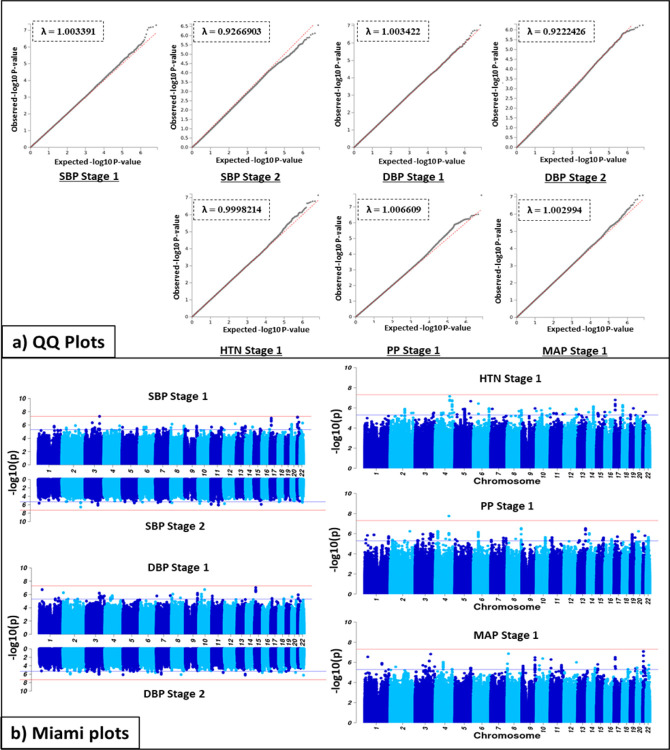
Discovery GWAS genetic associations in AWI-Gen (Stage 1) and the meta-analysis (Stage 2) showed by (a) Q-Q plots with the genomic control coefficient (λ) and (b) Miami plots for five BP traits. Adjusting for age, age2, sex and the first 10 PCs as covariates. With GW significance = p < 5E-8. QQ-plot shows the distribution of −log10-transformed p-value for observed (y-axis) vs expected (x-axis) with GIF (λ); red line = observed; grey line = expected. Miami plot shows −log10-transformed two-tailed p-value for each BP trait (y-axis) and base pair positions along the chromosomes (x-axis); red line = GW significance (p < 5E-08); purple line = threshold for suggestive association (p <1E-06).

**Figure 3:**
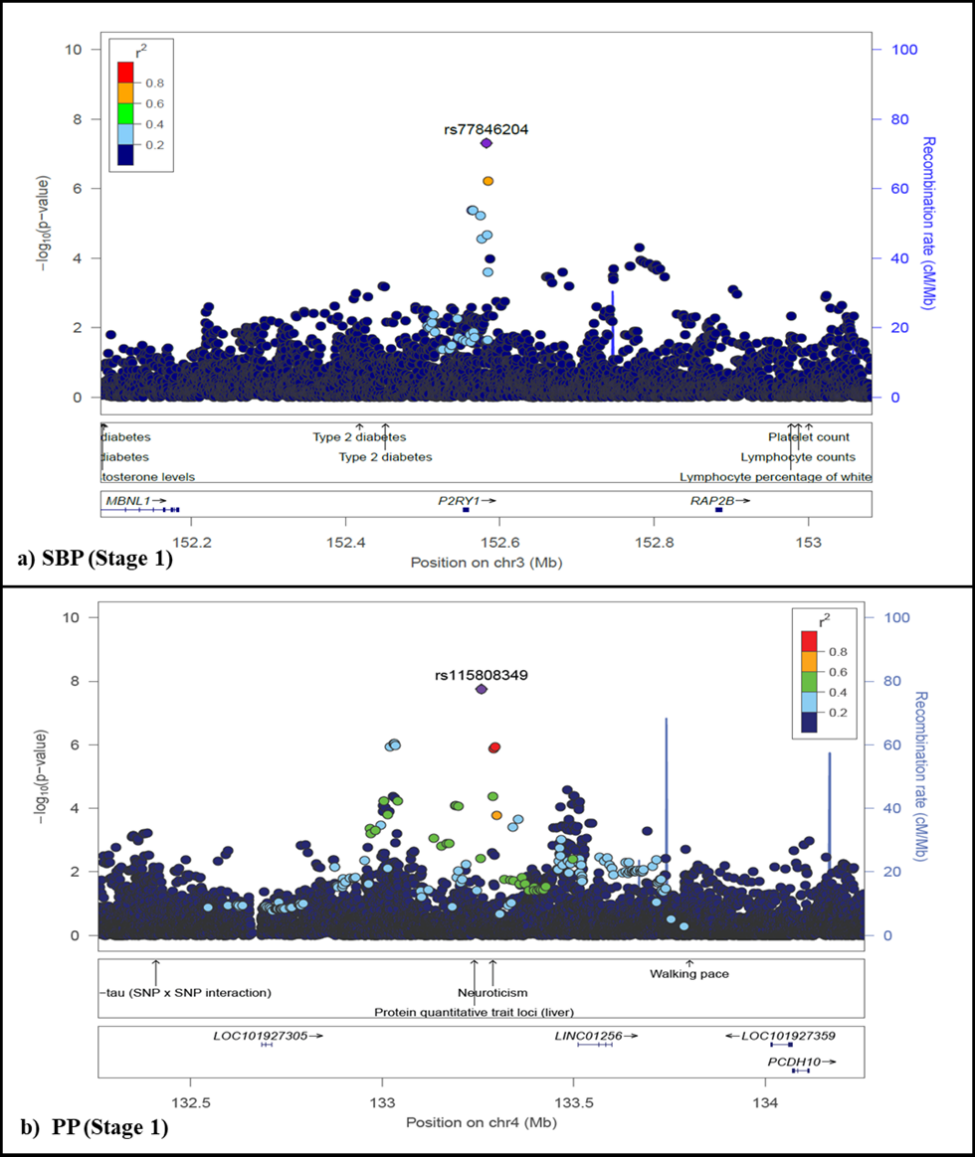
Fine mapping of novel GW significant (p < 5E-08) associations for BP-related traits in the AWI-Gen study. Locuszoom^73^ plots showing association for GW significant associations. Lead SNPs (purple diamond), GWAS Catalog trait labels and genes are labelled. Plots shown for SBP around the *P2RY1* region (rs77846204, p = 4.95E-08, intergenic *RP11–38P22.2*) and PP around the *Linc01256* region (rs115808348, p =1.76E-08,intergenic *ELL2P2* – also consisting of rs62317311 (p = 8.92E-07, ncRNA_intronic *RP11–789C2.1)*, for the AWI-Gen Stage 1 GWAS (N =10,775).

**Figure 4:**
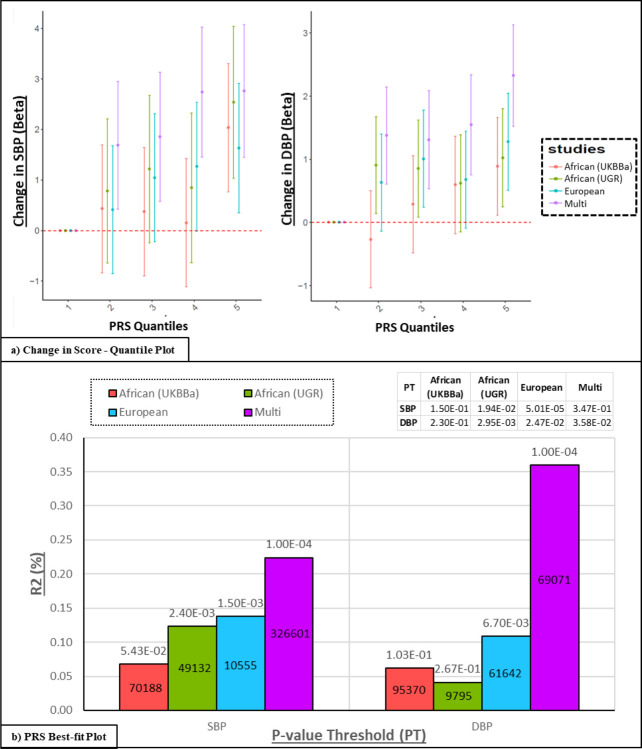
Transferability of Polygenic Risk Score (PRS). Models derived from three ancestry GWASs (discovery) and applied to the AWI-Gen cohort (target): (1) African: Two African-ancestry cohorts i.e. UKBBa (n=3,058) and UGR (n = 6,400)^46^ (2) European: UK biobank and ICPBP (N = 757,601)^22,41^ and (3) Multi-ancestry: PAGE (N = 49,839 with 17,152 African-ancestry) cohort^40,41^. a) PRS stratification of SBP and DBP: Point range-plots comparing the difference in BP-trait mean (mmHg) of the upper PRS quintiles from the lowest, stratified by the discovery datasets (error bars = mean ± 95% confidence intervals) are shown. b Plots showing additional variance explained (% R2) by each PRS for SBP and DBP: P-value (above bar) and number of SNPs (within bar) are stated for the P-threshold value (PT).

The summary workflow is shown in Figure 1.

### Ethics statement and consent

Ethical approval was obtained from the Human Research Ethics Committee (HREC) (Medical) of the University of the Witwatersrand (Protocol Number: M190927). This was a sub-study to the AWI-Gen study (Protocol Numbers: M121029, M170880, M2210108). Each of the participating sites also obtained ethics approval from their respective ethics committees. AWI-Gen sample data was used as permitted by the informed consent provided by the study participants and according to the H3Africa policies and guidelines (www.h3africa.org).

### Study participants

Participants were from the AWI-Gen study (10,775 participants, with the majority (89.3 %) aged between 40–60 years), located in three African regions (East, West, South) from six sites within four countries (Figure 1) i.e. East - Kenya (Nairobi); West - Burkina Faso (Nanoro) and Ghana (Navrongo); and South - South Africa (Agincourt, Dikgale, Soweto). Exclusion criteria for the study were: pregnant women, close relatives of existing participants (first and second-degree relatives), recent immigrants (who migrated <10 years ago into the region) and individuals with physical impairments preventing measurement of BP Further study cohort details can be found in Ramsay, et al.^35^ and Ali, et al.^36^.

Singh, et al.^47^ was used as a reference to identify previous GWAS for BP related traits in African populations, identifying only one study with summary statistics for SBP and DBP in a continental African population i.e. Gurdasani, et al.^46^.

### BP measurements

The outcome variables were SBP, DBP, HTN, PP and MAP. A digital sphygmomanometer (Omron M6, Omron, Kyoto, Japan) was used for BP measurements, which were taken three times at 2-minute intervals, with the last two measurements used to calculate the average SBP and DBP levels. PP and MAP were measured and calculated as continuous traits i.e. PP was calculated as the difference between the SBP and DBP and MAP was calculated as the sum of DBP and a third of the PP HTN (binary trait) was classified according to the Seventh Report of the Joint National Committee on Prevention, Detection, Evaluation, and Treatment of High BP (JNC7) guidelines^64^ (see Table S 1).

Hypertensive individuals (3683 cases; 6018 controls) were defined by the following conditions: individuals previously diagnosed with HTN and/or individuals taking medication for HTN and/or individuals having either SBP equal/above 140 mmHg or DBP equal/above 90 mmHg^10^. Adjustments were made for those taking AHM where 15mmHg were added to SBP and 10mmHg were added to DBP (N=2,293), as done in previous African studies^26,39,40^.

QC was performed on the phenotype data using Stata V15 (StataCorp, College Station, Texas, 77845, US)^65^ to assess outliers and distribution (Figure S 1).

### Genetic data and imputation

#### QC

Genotype data of ~11,000 samples was generated on the 2.3M SNP H3Africa genotyping array designed to include common African variants (https://chipinfo.h3abionet.org). The H3ABioNet/H3Agwas QC pipeline workflow^66^ (https://github.com/h3abionet/h3agwas/tree/master/qc) was used to conduct QC analysis as follows: (1) SNPs with high missingness (>0.02), low minor allele frequency (MAF) (<0.01), and extreme deviation from Hardy Weinberg Equilibrium proportions (HWE) (<0.0005) were excluded; (2) Samples with high genotype missingness (>0.01) and discordant sex information were removed; (3) Mitochondrial, Y and X chromosome SNPs, including SNPs that did not match the Genome Reference Consortium Human Build 37 (GRCh37/hg19) reference alleles were removed. After QC ~1.7 million SNPs and 10,903 samples remained^56^.

#### Imputation

Genotype imputation was conducted to increase the coverage of genomic variation and allow fine-mapping. The African Genome Resources reference panel (EAGLE2+PBWT pipeline) at the Sanger Imputation Server (https://imputation.sanger.ac.uk) was used for genotype imputation to increase the coverage of the genome, to narrow-down the location of potential causal variants and to capture most haplotype blocks. Post imputation QC (i.e. removal of indels, rare SNPs) resulted in 13,976,041 SNPs (MAF >0.01 and info score >0. 6)^56^.

Only participants with good quality phenotype and genotype data were used for the GWAS analyses (N=10,775). The genome assembly (base pair position) was the GRCh37/hg19.

### Genetic association analysis

The discovery GWAS for the BP-related traits was conducted in two stages (Figure 1).

#### Discovery GWAS (Stage 1 AWI-Gen GWAS)

Potential confounders used as covariates in the GWAS were examined for significance by running a general linear model, using STATA V15^65^. As the participants originate from East, West and Southern Africa, there was significant population structure across regions (Figure S 2); moreover, preliminary analysis indicated relatedness among individuals from some of the AWI-Gen cohorts. Therefore, adjustments based on PCs (addressing genetic population structure) and kinship-matrix (addressing relatedness) was used as covariates. Previously defined confounders were also used as covariates, using Singh, et al.^47^ as a guideline to determine adjustments (with exception to BMI which was not adjusted for in the previous studies which were included in the Stage 2 GWAS). All genetic association tests were adjusted for the covariates: age, age^2^, sex and the first 10 PCs (population structure and geographic region-based adjustments).

The H3ABioNet/H3Agwas Association pipeline workflow^66^ was used to conduct the discovery GWAS (https://github.com/h3abionet/h3agwas/). Novel associations were defined using the GWAS significant threshold significance of p<5E-08, with a suggestive threshold of p<5E-06. Linear mixed models (LMMs) were used to account for fixed and random effects for relatedness. Matrix LMMs were run to test for genetic associations, for an additive genetic model, for four continuous BP traits (SBP, DBP, PP and MAP) and one binary trait (HTN), using the Bayesian LMM association testing approach in BOLT-LMM v2.3.2 mixed model association testing^67^. This approach accounts for relatedness, ancestral heterogeneity (in samples) and any other unaccounted structure within the data.

Independent GWASs for each AWI-Gen region (East, West, South) was conducted and a meta-analysis of summary statistics was conducted, using RE2 (Han and Eskin’s Random Effects model), in METASOFT v2.0.1^68^. This was implemented in H3ABioNet/H3Agwas Meta-analysis pipeline workflow^66^ (http://github.com/h3abionet/h3agwas/meta/meta.nf), to evaluate the robustness of associations detected in joint analysis of the AWI-Gen dataset (Stage 1 GWAS). This approach assumes no heterogeneity of effect sizes if the null hypothesis is true (i.e. all beta values are zero), thus correcting for the overly conservative standard RE2 meta-analysis approach.

#### Power calculation

A power calculation was conducted (study design = continuous trait - independent individuals, hypothesis = gene-interaction, fixed number of samples = 10,903), using Quanto V1. 2. 3^69^. A graph for power versus effect size (beta) at different allele frequencies (see Figure S 3) was constructed in R^70^.

#### Meta-analysis (Stage 2 GWAS)

Previous studies, including only African and African-ancestry participants were used for a meta-analysis (sub-population of African participants from the UKBB (https://biobank.ctsu.ox.ac.uk) and Gurdasani, et al.^46^), to combine with the Stage 1 AWI-Gen meta-analysis GWAS, to improve study power (Stage 2). Permission was obtained to access the genotype and phenotype dataset of the UKBB (research project number: 63215). The UKBBa (N=3,060) was previously QCed and imputed, following the same methodology used for the Stage 1 GWAS.

The H3ABioNet/H3Agwas Association pipeline workflow^66^ was used to conduct the discovery GWAS (https://github.com/h3abionet/h3agwas/tree/master/assoc) for UKBBa, following the same methodology used for the Stage 1 GWAS. Gurdasani, et al.^46^ consisted of four African-ancestry cohorts: UGR (N=6,400), DDS (N=1,165), DCC (N=1,542) and AADM (N=5,231). Diabetes causes the walls of the blood vessels to stiffen, which leads to high BP^49–51^, therefore AADM, DDS and DCC, which included ~50% diabetic participants, were excluded.

The H3ABioNet/H3Agwas Meta-analysis pipeline workflow^66^ was implemented to conduct the Stage 2 meta-analysis (https://github.com/h3abionet/h3agwas/tree/master/meta). The meta-analysis was conducted for SBP and DBP by comparing the Stage 1 GWAS (meta-analysis of AWI-Gen GWAS by region) with the UKBBa dataset (N=3,058) and the UGR cohort (N=6,400)^46^ summary statistics, using RE2 in METASOFT v2.0.1^68^. Other BP-related traits (HTN, PP MAP) could not be included due to the lack of data availability in cohorts used in the Stage 2 GWAS.

#### Visualization and interpretation of genetic associations

Miami plots were generated, to display significantly associated SNPs in associated regions, using the Hudson package in R^71^. Genomic control and quantile-quantile (Q-Q) plots were conducted as a QC check, to re-evaluate genetic inflation and confounding biases such as cryptic relatedness and population stratification (with the assumption that the regional groupings will be independent of each other). Genomic control was evaluated in R^70^, by calculating GIF as, i.e. X^2^ = chi-squared of observed and 0.456 = median chi-squared of expected, where λ= 1 means that the population is homogenous and λ > 1.05 means that correction for population structure was not done efficiently and needs to be re-corrected. Q-Q plots were constructed in FUMA^72^ to assess deviation in the distribution of expected and observed p-values. Pleiotropy was identified when the same SNP meet suggestive significance (p<5E-06) in more than one BP-related trait.

Regional visualisation of associated SNP regions were performed using LD from AWI-Gen in LocusZoom V0.4.8^73^, using a 1MB flanking region, which was compared to data in the GWAS Catalog^17^.

### Replication with previous findings

Replication of the Stage 1 and 2 GWAS with populations of similar genetic ancestry was performed, using the exact replication strategy.^74^. Replication was also tested against the Stage 1 and 2 GWAS, using previous studies: (1) GWAS Catalog^17^; (2) PhenoScanner^37^; (3) List of all 3,800 published BP associated SNPs^38^; (4) European-only ancestry population from Evangelou, et al.^22^, consisting of the UKBB & ICPBP cohorts (currently the largest published study with 757,601 European ancestry individuals). Any duplicate signals from the same study across the previous study databases were removed.

Replication of the Stage 1 and 2 GWAS with previous studies, was assessed by comparing GW associations (p<5E-08) against SNPs with suggestive associations (p<5E-04). In addition, replication of GW signals found in previous studies (p<5E-08) were compared against the Stage 1 and 2 GWAS suggestive associations (p<5E-04).

The H3ABioNet/H3Agwas Replication pipeline workflow^66^ was implemented to conduct replication analysis (https://github.com/h3abionet/h3agwas/tree/master/replication). The exact replication method was used to test for replication with the GWAS Catalog. The GWAS Catalog database was downloaded (https://www.ebi.ac.uk/gwas/, accessed on 27 March 2022). Since the genome assembly of GWAS Catalog was the Genome Reference Consortium Human Build 38 (GRCh38/hg38), it was converted to GRCh37/hg19, to allow for comparison, by conducting a lift-over. A subset of the GWAS Catalog data was generated by filtering for key words relevant to BP traits: “Systolic blood pressure”, “Diastolic blood pressure”, “hypertension”, “pulse pressure”, and “mean-arterial pressure”.

The GW associations (p<5E-08) were also compared against the PhenoScanner database^37^, since the GWAS Catalog is limited to GW signals with p<5E-8 with a few suggestive at p<5E-06, to pick up any missed or additional suggestive signals (p<5E-04) found within this database (http://www.phenoscanner.medschl.cam.ac.uk). Replication of the Stage 1 and 2 GWAS was also compared against a list of 3,800 published BP associated SNPs listed within Warren, et al.^38^.

Exact replication was tested by using Evangelou et al. (2018) summary full statistics data (currently the largest published study with 757,601 European ancestry individuals), for SBP, DBP and PP to determine which signals were uniquely identified in studies with African-ancestry populations. Since both genome assemblies were GRCh37/hg19, a direct comparison was evaluated in R^70^.

Novel associated SNPs were determined by searching for SNPs within a 500 kb region of all SNPs with GW associations (p-value<5E-08) found in our study. The different regions of the AWI-Gen datasets were compared for South, East and West Africa (which were found to be significantly different sub-populations).

### *In silico* functional analysis

FUMA^72^ was used for *in silico* functional analysis and annotation was performed to select the most likely causal variants from the GWAS summary statistics. The FUMA pipeline (https://fuma.ctglab.nl) was used for functional gene mapping, using SNP2GENE tool, for positional, expression quantitative trait loci (eQTL) and chromatin interaction mappings. Candidate SNPs were selected in the associated genomic region with R2 ≥ 0.6 to define independent significant SNPs with GW (p < 5E-08) and MAF ≥ 01 for annotation (reference population = 1000G Phase3 African; included variants in reference panel (non-GWAS tagged SNPs in LD); maximum distance between LD blocks to merge into a locus = 250kb). Candidate SNPs functional consequences were predicted by chromosome base-pair position, and reference and alternate alleles, to databases containing known functional annotations. This included: (1) ANNOVAR, a variant annotation tool which is used to obtain functional consequences of SNPs on gene functions^75^; (2) combined annotation-dependent depletion (CADD), a score of deleteriousness of SNPs predicted by 63 functional annotations with a threshold of > 12.37 to be deleterious^76^; (3) RegulomeDB (RDB), a categorical score representing regulatory functionality of SNPs based on expression quantitative trait loci (eQTLs) and chromatin marks^77^, with eQTLs scans using the Genotype-Tissue Expression (GTex) Consortium^78^.

### Fine-mapping

Fine-mapping to identify potential causal variants was conducted by comparing the GW associations and/or top association SNPs where only suggestive associations were identified (lowest p<value SNP per trait), found in Stage 1 and 2 GWAS with previously reported BP loci. The H3ABioNet/H3Agwas Finemaping pipeline workflow^66^ was used for fine-mapping (https://github.com/h3abionet/h3agwas/tree/master/finemapping), which employs Bayesian calculation of posterior probability and/or annotation information to identify potential causal variants, using FINEMAP^79^.

### Polygenic risk score (PRS)

The PRS analysis was conducted using trait-specific effect-weighted variants obtained from the discovery GWAS. PRSice-2 V2.3.5^80^, a PRS software, was used to calculate and interpret PRSs. These steps included: (1) clumping (LD adjustment) to identify and select the most significant SNP in each LD block for further analyses; (2) p-value threshold (shrinkage strategy) to remove very low or non-significantly associated SNPs by performing multiple PRS analyses with varying p-value, and (3) plotting PRS results (Clumping distance = 250kb, R2 = 0.1, P- value thresholds for 1 to 5E-8).

The AWI-Gen study genotype data was used as the target database and models were derived from GWAS summary statistics for only SBP and DBP (due to BP-related trait data availability) from previous study groups by ancestry. Previous studies included: (1) African-ancestry only: A meta-analysis was conducted with the UKBBa dataset (N=3,058) and the UGR cohort (N=6,400)^46^ summary statistics, using the Han-Eskin random effects (RE2) model in METASOFT v2.0.1^68^, for the PRS African-ancestry only dataset; (2) European-ancestry only: Evangelou, et al.^22^ summary statistics, which consisted of UKBB and ICPBP cohorts (N=757,601), was used for the PRS European-ancestry only dataset; (3) Wojcik, et al.^41^ summary statistics, which consisted of the Population Architecture using Genomics and Epidemiology (PAGE) (N=49,839 with 17,152 AA) cohort, was used for the overall multi-ancestry dataset (AA, Hispanic/Latino, Asian-ancestry, Native Hawaiian-ancestry, Native American-ancestry).

The prediction models were adjusted for age, age2, sex and the first 10 PCs (population structure and geographic region based adjustments). The best predictive PRS were estimated using highest R^2^ (variance) and lowest p-values. The risk stratification of PRS were evaluated using quintile plots (comparing the difference in the mean of the phenotypic trait between the upper and lowest deciles).

## Data Availability

All data that support the findings of this study are available from the corresponding authors on request. The AWI-Gen data set is available from the European Genome-phenome Archive (EGA) database (https://ega-archive.org/), accession number EGAS00001002482 (phenotype dataset: EGAD00001006425; genotype dataset: EGAD00010001996). The availability of these datasets are subject to controlled access through, the Data and Biospecimen Access Committee of the H3Africa Consortium. The processed data generated in this study are provided in Supplementary Material. Permission was obtained to access the genotype and phenotype dataset for UKBB (research project number: 63215) (as described in Methods). Publicly available databases include (1) GWAS Catalog^17^ (https://www.ebi.ac.uk/gwas/), (2) PhenoScanner^37^ (http://www.phenoscanner.medschl.cam.ac.uk/). Other summary statistics reported in the paper are accessible on GWAS Catalog (https://www.ebi.ac.uk/gwas/) for (1) Gurdasani, et al.^46^ African-ancestry UGR cohort (SBP:GCST009053, DBP:GCST009052). (2) Evangelou, et al.^22^ European-ancestry UKBB & ICPBP cohorts (SBP: GCST006624, DBP: GCST006630, PP: GCST006629). (3) Wojcik, et al.^41^ multi-ancestry PAGE cohort (SBP: GCST008044, DBP: GCST008029).

## Abbreviations

1000G: 1000 Genome Project
AADM: Africa America Diabetes Mellitus Study
AWI-Gen: Africa Wits-INDEPTH partnership for Genomic Studies
AA: African American
AHM: Anti-hypertension medication
BP: Blood pressure
BMI: Body mass index
CVD: Cardiovascular disease
CADD: Combined annotation-dependent depletion
DBP: Diastolic blood pressure
DCC: Durban Case Control Study
DDS: Durban Diabetes Study
EGA: European Genome-phenome Archive
eQTLs: Expression quantitative trait loci
GRCh37/hg19: Genome Reference Consortium Human Build 37
GRCh38/hg38: Genome Reference Consortium Human Build 38
GW: Genome-wide
GWAS: Genome-wide association study
GIF: Genomic inflation factor
GTex: Genotype-Tissue Expression
RE2: Han-Eskin random effects
HWE: Hardy Weinberg Equilibrium proportions
H3Africa: Human Heredity and Health in Africa
HREC: Human Research Ethics Committee
HTN: Hypertension
ICBP: International Consortium of Blood Pressure
λ: Lambda
LMMs: Linear mixed models
LD: Linkage disequilibrium
MAP: Mean-arterial pressure
MAF: Minor allele frequency
PRS: Polygenic risk score
PAGE: Population Architecture using Genomics and Epidemiology
PCs: Principal components
PP: Pulse pressure
QC: Quality control
Q-Q: Quantile-quantile
RDB: RegulomeDB
JNC7: Seventh Report of the Joint National Committee on Prevention, Detection, Evaluation, and Treatment of High BP
SNPs: Single nucleotide polymorphisms
SBP: Systolic blood pressure
UGR: Uganda Genome Resource
UKBB: UK Biobank
UKBBa: UKBB African-ancestry
